# A Phylodynamic Workflow to Rapidly Gain Insights into the Dispersal History and Dynamics of SARS-CoV-2 Lineages

**DOI:** 10.1093/molbev/msaa284

**Published:** 2020-11-03

**Authors:** Simon Dellicour, Keith Durkin, Samuel L Hong, Bert Vanmechelen, Joan Martí-Carreras, Mandev S Gill, Cécile Meex, Sébastien Bontems, Emmanuel André, Marius Gilbert, Conor Walker, Nicola De Maio, Nuno R Faria, James Hadfield, Marie-Pierre Hayette, Vincent Bours, Tony Wawina-Bokalanga, Maria Artesi, Guy Baele, Piet Maes

**Affiliations:** 1 Spatial Epidemiology Lab (SpELL), Université Libre de Bruxelles, Bruxelles, Belgium; 2 Laboratory of Clinical and Epidemiological Virology, Department of Microbiology, Immunology and Transplantation, Rega Institute for Medical Research, KU Leuven, Leuven, Belgium; 3 Department of Human Genetics, CHU Liège, and Medical Genomics, GIGA Research Center, University of Liège, Liège, Belgium; 4 Department of Clinical Microbiology, University of Liège, Liège, Belgium; 5 European Molecular Biology Laboratory, European Bioinformatics Institute, Wellcome Genome Campus, Hinxton, Cambridgeshire, United Kingdom; 6 Department of Zoology, University of Oxford, Oxford, United Kingdom; 7 MRC Centre for Global Infectious Disease Analysis, J-IDEA, Imperial College London, London, United Kingdom; 8 Vaccine and Infectious Disease Division, Fred Hutchinson Cancer Research Center, Seattle, WA

**Keywords:** COVID-19, SARS-CoV-2, phylodynamic, phylogeography, phylogenetic clusters, lockdown measures

## Abstract

Since the start of the COVID-19 pandemic, an unprecedented number of genomic sequences of SARS-CoV-2 have been generated and shared with the scientific community. The unparalleled volume of available genetic data presents a unique opportunity to gain real-time insights into the virus transmission during the pandemic, but also a daunting computational hurdle if analyzed with gold-standard phylogeographic approaches. To tackle this practical limitation, we here describe and apply a rapid analytical pipeline to analyze the spatiotemporal dispersal history and dynamics of SARS-CoV-2 lineages. As a proof of concept, we focus on the Belgian epidemic, which has had one of the highest spatial densities of available SARS-CoV-2 genomes. Our pipeline has the potential to be quickly applied to other countries or regions, with key benefits in complementing epidemiological analyses in assessing the impact of intervention measures or their progressive easement.

First reported in early December 2019 in the province of Hubei (China), COVID-19 (coronavirus disease 2019) is caused by a new coronavirus (severe acute respiratory syndrome coronavirus 2; SARS-CoV-2) that has since rapidly spread around the world (Zhou et al. 2020; Zhu et al. 2020), causing an enormous public health and socioeconomic impact (Holmes et al. 2020; McKee and Stuckler, 2020). Since the early days of the pandemic, there has been a necessary mobilization of the scientific community to understand its epidemiology and provide a real-time response. To this end, research teams around the world have massively sequenced and publicly released dozens of thousands of viral genome sequences to study the origin of the virus (Andersen et al. 2020; Lu et al. 2020), and to trace its spread at global, country or community-level scales (Eden et al. 2020; Lu et al. 2020; Rivett et al. 2020). In this context, a platform like Nexstrain (Hadfield et al. 2018), already widely used and recognized by the academic community and beyond, has quickly become a reference to follow the dispersal history of SARS-CoV-2 lineages.

In the context of the COVID-19 pandemic, the volume of genomic data available presents a unique opportunity to gain valuable real-time insights into the dispersal dynamics of the virus. However, the number of available viral genomes is increasing every day, leading to substantial computational challenges. Although Bayesian phylogeographic inference represents the gold standard for inferring the dispersal history of viral lineages (Baele et al. 2018), these methods are computationally intensive and may fail to provide useful results in an acceptable amount of time for large data sets. To tackle this practical limitation, we here describe and apply an analytical pipeline that is a compromise between fast and rigorous analytical steps. In practice, we propose to take advantage of the rapid time-scaled phylogenetic tree inference process used by the online Nextstrain platform (Hadfield et al. 2018). Specifically, we aim to use the resulting time-scaled tree as a fixed empirical tree along which we infer the ancestral locations with the discrete (Lemey et al. 2009) and spatially explicit (Lemey et al. 2010) phylogeographic models implemented in the software package BEAST 1.10 (Suchard et al. 2018).

In Belgium, there are two main laboratories (from the University of Leuven and the University of Liège) involved in sequencing SARS-CoV-2 genomes extracted from confirmed COVID-19 positive patients. To date, some genomes (*n *=* *58) have also been sequenced at the University of Ghent, but for which metadata about the geographic origin are unavailable. As of June 10, 2020, a total of 740 genomes have been sequenced by these research teams and deposited in the GISAID (Global Initiative on Sharing All Influenza Data [Shu and McCauley, 2017]) database. In the present study, we exploit this comprehensive data set to uncover the dispersal history and dynamics of SARS-CoV-2 viral lineages in Belgium. In particular, our objective is to investigate the evolution of the circulation dynamics through time and assess the impact of lockdown measures on spatial transmission. Specifically, we aim to use phylogeographic approaches to look at the Belgian epidemic at two different levels: 1) the importance of introduction events into the country, and 2) viral lineages circulating at the nationwide level. Our analytical pipeline is detailed in Supplementary Information.

## Results

### Importance of Introduction Events into the Country

**Fig. 1:**
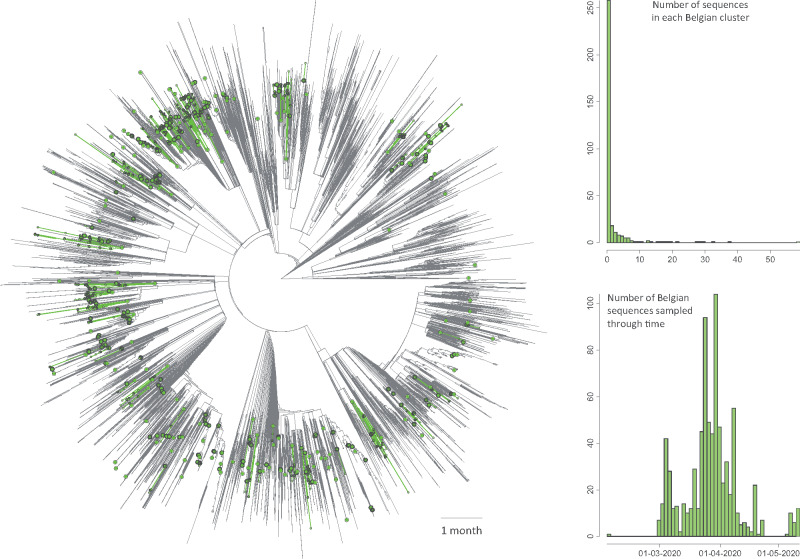
Time-scaled phylogenetic tree in which we identified Belgian clusters. A cluster is here defined as a phylogenetic clade likely corresponding to a distinct introduction into the study area (Belgium). We delineated these clusters by performing a simplistic discrete phylogeographic reconstruction along the time-scaled phylogenetic tree while only considering two potential ancestral locations: “Belgium” and “non-Belgium”. We identified a minimum number of 331 lineage introductions (95% HPD interval = [315–344]), which showcases the relative importance of external introductions considering the number of sequences currently sampled in Belgium (*n = *740). On the tree, lineages circulating in Belgium are highlighted in green, and green nodes correspond to the most ancestral node of each Belgian cluster (see also supplementary fig. S1, Supplementary Material online for a noncircular visualization of the same tree). Besides the tree, we also report the distribution of cluster sizes (number of sampled sequences in each cluster) as well as the number of sequences sampled through time.

On June 10, 2020, we downloaded all Belgian SARS-CoV-2 sequences (*n *=* *740) available on GISAID, as well as all non-Belgian sequences used in Nextstrain to represent the overall dispersal history of the virus (*n *=* *4,309 with 126 different countries of origin). We generated a time-scaled phylogenetic tree using a rapid maximum likelihood approach (Sagulenko et al. 2018) and subsequently ran a preliminary discrete phylogeographic analysis along this tree to identify internal nodes and descending clades that likely correspond to distinct introductions into the Belgian territory (fig. 1, supplementary fig. S2, Supplementary Material online). We inferred a minimum number of 331 introduction events (95% HPD interval = [315–344]). When compared with the number of sequences sampled in Belgium (*n *=* *740), this number illustrates the relative importance of external introductions in establishing transmission chains in the country. Introduction events resulted in distinct clades (or “clusters”) linking varying numbers of sampled sequences (fig. 1). However, many clusters only consisted of one sampled sequence. According to the time-scaled phylogenetic tree and discrete phylogeographic reconstruction (supplementary fig. S1, Supplementary Material online), some of these introduction events could have occurred before the return of Belgian residents from carnival holidays (around March 1, 2020), which was considered to be the major entry point in time of transmission chains in Belgium.

### Impact of Lockdown Measures at the Country Level

**Fig. 2:**
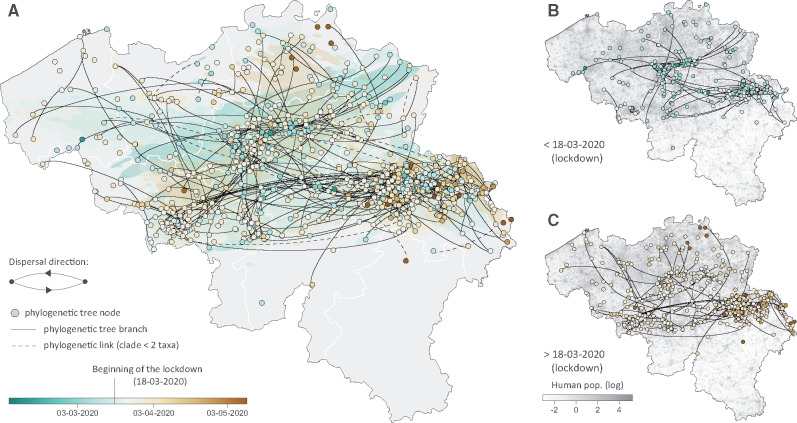
Spatially explicit phylogeographic reconstruction of the dispersal history of SARS-CoV-2 lineages in Belgium. (*A*) Continuous phylogeographic reconstruction performed along each Belgian clade (cluster) identified by the initial discrete phylogeographic analysis. For each clade, we mapped the maximum clade credibility (MCC) tree and overall 80% highest posterior density (HPD) regions reflecting the uncertainty related to the phylogeographic inference. MCC trees and 80% HPD regions are based on 1,000 trees subsampled from each post burn-in posterior distribution. MCC tree nodes were colored according to their time of occurrence, and 80% HPD regions were computed for successive time layers and then superimposed using the same color scale reflecting time. Continuous phylogeographic reconstructions were only performed along Belgian clades linking at least three sampled sequences for which the geographic origin was known (see the detailed analytical pipeline in Supplementary Information for further detail). Besides the phylogenetic branches of MCC trees obtained by continuous phylogeographic inference, we also mapped sampled sequences belonging to clades linking less than three geo-referenced sequences. Furthermore, when a clade only gathers two geo-referenced sequences, we highlighted the phylogenetic link between these two sequences with a dashed curve connecting them. Subnational province borders are represented by white lines. (*B*) MCC tree branches occurring before March 18, 2020 (beginning of the lockdown). (*C*) MCC tree branches occurring after March 18, 2020. See also supplementary figure S2, Supplementary Material online, for a zoomed version of the dispersal history of viral lineages in the Province of Liège, for which we have a particularly dense sampling.

To analyze the circulation dynamics of viral lineages within the country, we then performed spatially explicit phylogeographic inference along the previously identified Belgian clades (fig. 2*A*). Our reconstructions reveal the occurrence of long-distance dispersal events both before (fig. 2*B*) and during (fig. 2*C*) the lockdown. By placing phylogenetic branches in a geographical context, spatially explicit phylogeographic inference allows for treating those branches as conditionally independent movement vectors (Pybus et al. 2012). Here, we looked at these movement vectors to assess how the dispersal dynamics of lineages was impacted by the national lockdown, of which the main measures were implemented on March 18, 2020.

**Fig. 3:**
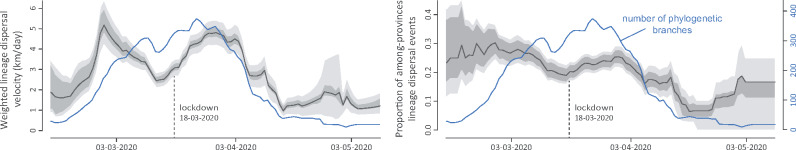
Evolution of viral lineage dispersal dynamics during the Belgian epidemic. These estimates are based on 1,000 trees subsampled from each post burn-in posterior distribution. Except for the number of phylogenetic branches occurring at each time slice, all estimates were smoothed using a 14-days sliding window. Dark gray surrounding polygons represent 95% credible intervals, and light gray surrounding polygons represent 95% credible intervals re-estimated after subsampling 75% of branches in each of the 1,000 posterior trees. The credible interval based on the subsampling procedure is an indication of the robustness of the estimate. In addition, we also report the number of phylogenetic branches occurring per tree at each time slice (blue curve). The number of branches available at each time slice is an additional, yet qualitative, indication of robustness of the estimate for a given time period.

Firstly, we investigated whether the national lockdown was associated with a change in lineage dispersal velocity. We estimated a higher dispersal velocity before the lockdown (5.4 km/day, 95% HPD [5.0–6.1]) compared with during the lockdown (2.4 km/day, 95% HPD [2.3–2.5]). This trend is further confirmed when focusing on the Province of Liège for which we have a particularly dense sampling: in this province, we estimated a lineage dispersal velocity of 2.8 km/day (95% HPD [2.2–3.6]) before the lockdown and of 1.1 km/day (95% HPD [1.0–1.2]) during the lockdown. However, the evolution of the lineage dispersal velocity through time is less straightforward to interpret (fig. 3): although the lineage dispersal velocity was globally higher at the early phase of the Belgian epidemic, which corresponds to the week following the returns from carnival holidays, it then seemed to drop just before the beginning of the lockdown before increasing again to reach a plateau. In the second half of April, our estimates indicate that the lineage dispersal velocity drops again. However, this result may be an artefact associated with the lower number of phylogenetic branches currently inferred during that period (fig. 3).

Secondly, we further investigated the impact of the lockdown on the lineage dispersal events among provinces. Our analyses indicate that lineage dispersal events among provinces tended to decrease during the epidemic (fig. 3): such dispersal events were more frequent at the beginning of the epidemic and then progressively decreased until reaching a plateau at the beginning of the lockdown. Again, the relatively limited number of phylogenetic branches currently inferred from mid-April does not convincingly allow for interpretation of the fluctuations of the proportion of lineage dispersal events among provinces during that period.

Finally, because our approach is dependent on the initial time-scaled tree, we also assess the robustness of our results to the selection of the maximum likelihood starting tree. Specifically, we reran the analytical pipeline on five additional time-scaled trees independently obtained using maximum likelihood inference. We then compared the inferred number of introduction events into the Belgian territory as well as the lineage dispersal velocity estimates obtained when running the pipeline on each starting tree (supplementary table S1, Supplementary Material online). Despite some relatively low differences in lineage dispersal velocity estimates, our results confirmed that the different estimates remain robust to the choice of the starting tree and that our pipeline appears not to be substantially impacted by the statistical uncertainty associated with the initial maximum likelihood inference step.

## Discussion

Our phylogeographic investigation of the first wave of the SARS-CoV-2 epidemic in Belgium reveals the important contribution of external introduction events toward the establishment of the epidemic in the country. This highlights that transmission chains circulating in Belgium were not established by a relatively restricted number of isolated infectious cases. On the contrary, we identify a large number of distinct clades given the number of analyzed sequences sampled in Belgium. This overall observation is in line with other reports, for example, in California where no predominant lineage was identified either (Deng et al. 2020).

Our spatially explicit phylogeographic analyses uncover the spatiotemporal distribution of Belgian SARS-CoV-2 clusters, indicating a relatively low impact of the lockdown on both the dispersal velocity of viral lineages and on the frequency of long-distance dispersal events. Although it has been demonstrated that the national lockdown had an overall impact on the virus transmission, that is, reducing its effective reproduction number to a value below one (Coletti et al. 2020), our results highlight that the lockdown did not clearly decrease the velocity at which the viral lineages travelled or their ability to disperse over long distances within the country. This finding may be important to consider in the context of potential future lockdown measures, especially if more localized (e.g., at the province or city level). Indeed, locally reduced transmission rates will not automatically be associated with a notable decrease in the average velocity or distance travelled by lineage dispersal events, which could in turn limit the effectiveness of localized lockdown measures in containing local upsurge of the virus circulation.

Applying the present phylodynamic pipeline in a real-time perspective does not come without risk as new sequences can sometimes be associated with spurious nucleotide changes that could be due to sequencing or assembling errors. Directly starting from inference results kept up to date by a database like GISAID allows for fast analytical processing but also relies on newly deposited data that could sometimes carry errors. To remedy such potentially challenging situations, our proposed pipeline could be extended with a sequence data resource component that makes uses of expert knowledge regarding a particular virus. The GLUE (Singer et al. 2018) software package allows new sequences to be systematically checked for potential issues, and could hence be an efficient tool to safely work with frequently updated SARS-CoV-2 sequencing data. Such a “CoV-GLUE” resource is currently being developed (http://cov-glue.cvr.gla.ac.uk/#/home, last accessed November 13, 2020).

Although we acknowledge that a fully integrated analysis (i.e., an analysis where the phylogenetic tree and ancestral locations are jointly inferred) would be preferable, fixing an empirical time-scaled tree represents a good compromise to rapidly gain insights into the dispersal history and dynamics of SARS-CoV-2 lineages. Indeed, the number of genomes available, as well as the number of different sampling locations to consider in the analysis, would lead to a joint analysis requiring weeks of run-time in a Bayesian phylogenetic software package like BEAST. To illustrate the computational demands of such an approach, we ran a classic Bayesian phylogenetic analysis on a smaller SARS-CoV-2 data set (2,795 genomic sequences) using BEAST 1.10 (data not shown). This analysis required over 150 h to obtain enough samples from the posterior distribution, while using the latest GPU accelerated implementations (Ayres et al. 2019) across 15 parallel runs. With a combined chain length of over 2.2 × 10^9^ states, and an average runtime of 0.9 h per million states, the significant computational demands make this approach impractical when speed is critical. On the other hand, here we use a maximum likelihood method implemented in the program TreeTime (Sagulenko et al. 2018) to obtain a time-scaled phylogenetic tree in a short amount of time (∼3 h for the data set analyzed here). Given the present urgent situation, we have deliberately assumed a time-scaled maximum likelihood phylogenetic tree as a fair estimate of the true time-scaled phylogenetic tree.

Our analytical workflow has the potential to be rapidly applied to study the dispersal history and dynamics of SARS-CoV-2 lineages in other restricted or even much broader study areas. We believe that spatially explicit reconstruction can be a valuable tool for highlighting specific patterns related to the circulation of the virus or assessing the impact of intervention measures. Although new viral genomes are sequenced and released daily, a limitation could paradoxically arise from the nonaccessibility of associated metadata. Indeed, without sufficiently precise sampling location data for each genome, it is not feasible to perform a spatially explicit phylogeographic reconstruction. In the same way that viral genomes are deposited in databases like GISAID, metadata should also be made available to enable comprehensive epidemiological investigations using a similar approach as we presented here.

## Supplementary Material

Supplementary data are available at *Molecular Biology and Evolution* online.

## Supplementary Material

msaa284_Supplementary_Data
