# Supplementary material for: A Phylodynamic Workflow to Rapidly Gain Insights into the Dispersal History and Dynamics of SARS-CoV-2 Lineages
Source: Mol Biol Evol. 2020 Nov 3;38(4):1608–13. doi: 10.1093/molbev/msaa284 (PMC7665608; doi:10.1093/molbev/msaa284)
Supplement: msaa284_Supplementary_Data [file msaa284_supplementary_data.pdf]

Supplementary Information: detailed analytical pipeline

SARS-CoV-2 sequencing in Belgium

At the University of Leuven, RNA extracts from SARS-CoV-2 infected patients were selected anonymously and based on a city's postal code within Belgium. These RNA extracts were provided by the National Reference Center for Coronaviruses and UZ Leuven. Reverse transcription was carried out via SuperScript IV and cDNA was posteriorly amplified using Q5® High-Fidelity DNA Polymerase (NEB) with the ARTIC nCov-2019 primers and following the recommendations in the sequencing protocol of the ARTIC Network (https://artic.network/ncov-2019). Samples were multiplexed following the manufacturer's recommendations using the Oxford Nanopore Native Barcoding Expansion kits NBD104 (1-12) and NBD114 (13-24), in conjunction with Ligation Sequencing Kit 109 (Oxford Nanopore). Sequencing was carried out on a MinION sequencer using R9.4.1 flow cells and MinKNOW 2.0 software.

At the University of Liège, RNA was extracted from clinical samples (300µl) via a Maxwell 48 device using the Maxwell RSC Viral TNA kit (Promega) with a viral inactivation step using Proteinase K, following the manufacturer's instructions. RNA elution occurred in 50µL of RNAse free water. Reverse transcription was carried out via SuperScript IV VILOTM Master Mix, and 3.3 µl of the eluted RNA was combined with 1.2 µl of master mix and 1.5µl of H_2O. This was incubated at 25°C for 10 min, 50°C for 10 min and 85°C for 5 min. PCR used Q5® High-Fidelity DNA Polymerase (NEB), the primers and conditions followed the recommendations in the sequencing protocol of the ARTIC Network. Samples were multiplexed following the manufacturer's recommendations using the Oxford Nanopore Native Barcoding Expansion kits 1-12 and 13-24, in conjunction with Ligation Sequencing Kit 109 (Oxford Nanopore). Sequencing was carried out on a Minion using R9.4.1 flow cells. Data analysis followed the SARS-CoV-2 bioinformatics protocol of the ARTIC Network.

Inference of a time-scaled phylogenetic tree

To infer a time-scaled phylogenetic tree, we selected all non-Belgian sequences in the Nextstrain analysis, along with all available Belgian sequences in GISAID as of June 10, 2020, to be included in our analysis. Once we knew which were the accessions of interest, we downloaded the latest whole genome alignment from GISAID. We then cleaned the alignment by manually trimming the 5' and 3' untranslated regions (RefSeq NC_045512.2) and gap-only sites. To obtain a maximum likelihood phylogeny, we ran IQ-TREE 2.0.3 (Minh et al. 2020) under a general time reversible (Tavaré 1986) (GTR) model of nucleotide substitution with empirical base frequencies and four free rate (Yang 1995) site categories. This model configuration was selected as the best GTR model using IQ-TREE's ModelFinder tool. The tree was then inspected for outlier sequences using TempEst (Rambaut et al. 2016) 1.5.3 and, once the outliers were removed, time-calibrated using TreeTime 0.7.4 (Sagulenko et al. 2018). To replicate the Nextstrain workflow as closely as possible, we specified a clock rate of 8×10^{-4} in TreeTime and removed samples that deviate more than four interquartile ranges from the root-to-tip regression (https://github.com/nextstrain/ncov).

Preliminary discrete phylogeographic analysis

We performed a preliminary phylogeographic analysis using the discrete diffusion model (Lemey et al. 2009) implemented in the software package BEAST 1.10 (Suchard et al. 2018). The objective of this first analysis was to identify independent introduction events of SARS-CoV-2 lineages into Belgium. To this end, we used our time-scaled phylogenetic tree as a fixed empirical tree and only considered two possible ancestral locations: "Belgium" and "non-Belgium". Bayesian inference through Markov chain Monte Carlo (MCMC) was run on this empirical tree for 10^6 generations and sampled every 1,000 generations. MCMC convergence and mixing properties were inspected using the program Tracer 1.7 (Rambaut et al. 2018) to ensure that effective sample size (ESS) values associated with estimated parameters were all >200. After having discarded 10% of sampled trees as burn-in, a maximum clade credibility (MCC) tree was obtained using TreeAnnotator 1.10 (Suchard et al. 2018). We used the resulting MCC tree to delineate Belgian clusters here defined as phylogenetic clades corresponding to independent introduction events in Belgium. In practice, we identified introduction events by comparing the locations assigned to each pair of nodes connected by the phylogenetic branches of this MCC tree, i.e. the most probable location inferred at internal nodes and the sampling location for tip nodes. We considered an introduction event to be the case when the location assigned to a node was "Belgium" and the location assigned to its parent node in the tree was "non-Belgium".

Continuous and post hoc phylogeographic analyses

We used the continuous diffusion model (Lemey et al. 2010) available in BEAST 1.10 (Suchard et al. 2018) to perform a spatially-explicit (or "continuous") phylogeographic reconstruction of the dispersal history of SARS-

CoV-2 lineages in Belgium. We employed a relaxed random walk (RRW) diffusion model to generate a posterior distribution of trees whose internal nodes are associated with geographic coordinates (Lemey et al. 2010). Specifically, we used a Cauchy distribution to model the among-branch heterogeneity in diffusion velocity. We performed a distinct continuous phylogeographic reconstruction for each Belgian clade identified by the initial discrete phylogeographic inference, again fixing a time-scaled subtree as an empirical tree. As phylogeographic inference under the continuous diffusion model does not allow identical sampling coordinates assigned to the tips of the tree, we avoided assigning sampling coordinates using the centroid point of each administrative area of origin. For a given sampled sequence, we instead retrieved geographic coordinates from a point randomly sampled within its municipality of origin, which is the maximal level of spatial precision in available metadata. This approach avoids using the common "jitter" option that adds a restricted amount of noise to duplicated sampling coordinates. Using such a jitter could be problematic because it can move sampling coordinates to administrative areas neighbouring their actual administrative area of origin (Dellicour et al. 2018). Furthermore, the administrative areas considered here are municipalities and are rather small (there are currently 581 municipalities in Belgium). The clade-specific continuous phylogeographic reconstructions were only based on Belgian tip nodes for which the municipality of origin was known, i.e. 639 out of 740 genomic sequences. Furthermore, we only performed a continuous phylogeographic inference for Belgian clades linking a minimum of three tip nodes with a known sampling location (municipality).

Each Markov chain was run for 10^6 generations and sampled every 1,000 generations. As with the discrete phylogeographic inference, MCMC convergence/mixing properties were assessed with Tracer, and MCC trees (one per clade) were obtained with TreeAnnotator after discarding 10% of sampled trees as burn-in. We then used functions available in the R package "seraphim" (Dellicour et al. 2016) to extract spatiotemporal information embedded within the same 1,000 posterior trees and visualise the continuous phylogeographic reconstructions. We also used "seraphim" to estimate the following weighted lineage dispersal velocity:

$$v_{weighted} = \frac{\sum_{i=1}^{n} d_i}{\sum_{i=1}^{n} t_i}$$

where d_i and t_i are the geographic distance travelled (great-circle distance in km) and the time elapsed (in days) on each phylogeny branch, respectively. Weighted lineage dispersal velocity was estimated before and during the lockdown by selecting phylogeny branches for which both nodes occurred before or during the lockdown (using March 18, 2020, as a cut-off), respectively. Consequently, phylogeny branches with ancestral and youngest nodes occurring respectively before and during the lockdown were discarded for these estimates of lineage dispersal velocity. Furthermore, we also estimated the evolution of the weighted lineage dispersal velocity through time, computing estimates over a sliding window of two weeks allowing a minimum number of phylogeny branches to be considered in each time. The choice of this weighted metric was motivated by the low variance associated with its estimates, which results from a restricted contribution of phylogeny branches with short duration, making it particularly useful when aiming to compare different data sets or different time periods (Dellicour et al. 2019). Consequently, depending on the duration of branches overlapping each time slice, estimates obtained for successive time slices do not necessarily contribute equally to the overall estimate obtained for the entire period under consideration. We verified the robustness of our estimates through a subsampling procedure consisting of re-computing the weighted dispersal velocity after having randomly discarded 25% of branches in each of the 1,000 posterior trees.

Assessing the robustness of the pipeline to the selection of the starting tree

Because our approach is dependent on the time-scaled phylogenetic tree selected as a starting point of the pipeline, we performed an additional analysis to assess if our results are robust to the choice of that starting tree. Specifically, we performed five additional independent replicates of maximum likelihood phylogenetic inference in IQ-TREE using different starting seeds. We then re-ran our entire workflow using these alternative trees as starting points, which allowed us to investigate to what extent the topological uncertainty associated with the maximum likelihood inference step could have an impact on the different estimates. We reported and compared the results obtained when considering each starting tree (Table S1): the number of independent introduction events identified on the Belgian territory, and the weighted lineage dispersal velocity (before and during lockdown).

Table S1. Robustness test of the pipeline to the selection of the starting tree. We here report estimates (and associated 95% HPD intervals) obtained when running our pipeline on distinct starting phylogenetic trees independently inferred using IQ-TREE.

Time-scaled phylogenetic tree inference	Inferred number of introduction events in Belgium	Weighted lineage dispersal velocity (km/day)	
		before the lockdown	during the lockdown
Replicate #1	340 [328-349]	4.6 km/day [4.2-5.2]	2.2 km/day [2.1-2.3]
Replicate #2	322 [308-337]	4.3 km/day [3.9-4.8]	2.7 km/day [2.6-2.8]
Replicate #3	304 [289-319]	5.8 km/day [5.4-6.4]	2.3 km/day [2.3-2.5]
Replicate #4	336 [323-351]	4.3 km/day [3.9-4.9]	2.1 km/day [2.0-2.2]
Replicate #5	325 [313-337]	4.6 km/day [4.2-5.2]	2.3 km/day [2.2-2.3]

References

Dellicour S, Baele G, Dudas G, Faria NR, Pybus OG, Suchard MA, Rambaut A, Lemey P. 2018. Phylodynamic assessment of intervention strategies for the West African Ebola virus outbreak. *Nat. Commun.* 9:2222.

Dellicour S, Rose R, Faria NR, Lemey P, Pybus OG. 2016. SERAPHIM: studying environmental rasters and phylogenetically informed movements. *Bioinformatics* 32:3204–3206.

Dellicour S, Troupin C, Jahanbakhsh F, Salama A, Massoudi S, Moghaddam MK, Baele G, Lemey P, Gholami A, Bourhy H. 2019. Using phylogeographic approaches to analyse the dispersal history, velocity, and direction of viral lineages – application to rabies virus spread in Iran. *Mol. Ecol.* 28:4335–4350.

Lemey P, Rambaut A, Drummond AJ, Suchard MA. 2009. Bayesian phylogeography finds its roots. *PLoS Comput. Biol.* 5:e1000520.

Lemey P, Rambaut A, Welch JJ, Suchard MA. 2010. Phylogeography takes a relaxed random walk in continuous space and time. *Mol. Biol. Evol.* 27:1877–1885.

Minh BQ, Schmidt HA, Chernomor O, Schrempf D, Woodhams MD, von Haeseler A, Lanfear R. 2020. IQ-TREE 2: New models and efficient methods for phylogenetic inference in the genomic era. *Mol. Biol. Evol.* 37:1530–1534.

Rambaut A, Drummond AJ, Xie D, Baele G, Suchard MA. 2018. Posterior summarization in Bayesian phylogenetics using Tracer 1.7. *Syst. Biol.* 67:901–904.

Rambaut A, Lam TT, Max Carvalho L, Pybus OG. 2016. Exploring the temporal structure of heterochronous sequences using TempEst (formerly Path-O-Gen). *Virus Evol* [Internet] 2. Available from: https://www.ncbi.nlm.nih.gov/pmc/articles/PMC4989882/

Sagulenko P, Puller V, Neher RA. 2018. TreeTime: Maximum-likelihood phylodynamic analysis. *Virus Evol.* 4:vex042.

Suchard MA, Lemey P, Baele G, Ayres DL, Drummond AJ, Rambaut A. 2018. Bayesian phylogenetic and phylodynamic data integration using BEAST 1.10. *Virus Evol.* 4:vey016.

Tavaré S. 1986. Some probabilistic and statistical problems in the analysis of DNA sequences. *Lectures Math. Life Sci.* 17:57–86.

Yang Z. 1995. A space-time process model for the evolution of DNA sequences. *Genetics* 139:993–1005.

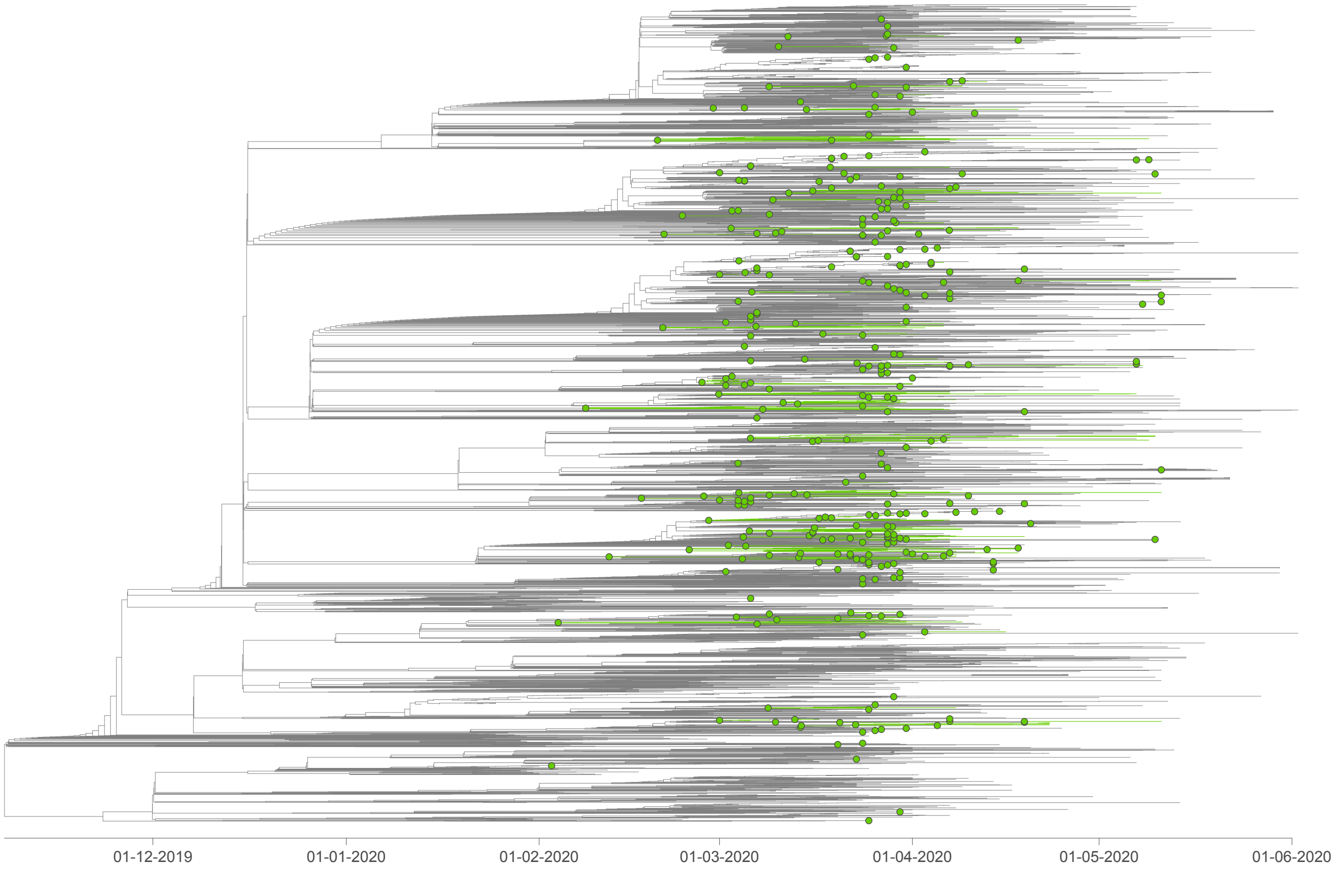

Figure S1. Time-scaled phylogenetic tree in which we identified Belgian clusters. A cluster is here defined as a phylogenetic clade likely corresponding to a distinct introduction into the Belgian territory. We delineated these clusters by performing a simplistic discrete phylogeographic reconstruction along the time-scaled phylogenetic tree while only considering two potential ancestral locations: "Belgium" and "outside Belgium". On the tree, lineages circulating in Belgium are highlighted in green, and green nodes correspond to the most ancestral node of each Belgian cluster.

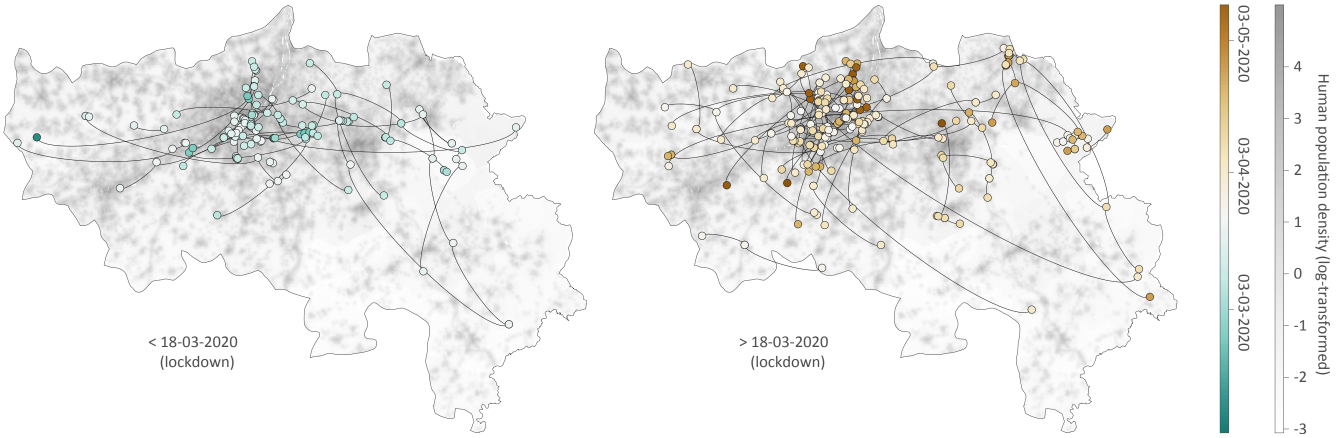

Figure S2. Spatially-explicit phylogeographic reconstruction of the dispersal history of SARS-CoV-2 lineages in the Province of Liège. Continuous phylogeographic reconstruction was performed along each Belgian clade (cluster) identified by the initial discrete phylogeographic analysis. For each clade, we mapped the maximum clade credibility (MCC) branches located in the province of Liège, before and after the 18th March 2020 (i.e. the beginning of the lockdown).
